# In memory of Professor Israel Silman (1935–2025)

**DOI:** 10.1002/pro.70191

**Published:** 2025-06-25

**Authors:** Joel L. Sussman

**Affiliations:** ^1^ Department of Chemical and Structural Biology Weizmann Institute of Science Rehovot Israel

Professor Israel Silman of the Weizmann Institute of Science passed away on February 26, 2025, just shy of his 90th birthday. “Sili,” as he was affectionately known to friends and colleagues, was a neurobiologist of rare depth and clarity, a scientist in the truest and most thoughtful sense. His foundational work on acetylcholinesterase's biochemistry and structural biology (AChE) reshaped the field and influenced generations of scientists.

Sili combined incisive scientific instincts with an old‐world sensibility. He believed that the most profound ideas often emerged not at the bench but in moments of quiet reflection. One of his most fertile periods of invention, as I came to learn, unfolded during our daily mid‐morning Turkish coffee breaks, a cherished ritual shared with Lev Weiner, Yacov Ashani, and me. Far from idle moments, these gatherings were crucibles of creativity, where critical experiments were first conceived, sometimes sketched on the back of a napkin.

Born in Shipley, UK, in 1935, Sili immigrated with his parents to Israel in 1950, settling in Jerusalem. After military service in the Israel Defense Forces, he pursued undergraduate and master's degrees in biochemistry and organic chemistry at the Hebrew University of Jerusalem. His doctoral research at the Weizmann Institute, under Professor Ephraim Katchalski (later Katzir), focused on enzyme immobilization, an early foray into his lifelong fascination with the interplay between molecular structure and function, and he received his PhD from the Hebrew University in 1964.

Sili formally joined the Weizmann Institute in 1964. A Fulbright‐Hays Fellowship took him to the University of Wisconsin and then to Columbia University's Department of Neurology, where he worked with Professor David Nachmansohn. There, he encountered acetylcholine receptors and acetylcholinesterase, the molecular pair that would define his scientific legacy.

Upon returning to Weizmann in 1968 and later as a founding member of the Department of Neurobiology in 1976, Sili built a vibrant research program. Prestigious fellowships, including those from EMBO and Minerva, supported his time abroad at institutions such as Imperial College London and the Max Planck Institute in Göttingen. Yet it was in Rehovot that Sili truly made his mark, with work characterized by biochemical rigor, molecular creativity, and relentless curiosity.

We first met serendipitously in 1984 during overlapping sabbaticals in the United States. Our wives, Aliza and Haya, had arranged a dinner. What began as a social evening quickly became a scientific courtship. Sili and I discovered a shared fascination with enzyme structure, particularly AChE and its cousin, chymotrypsin. The next day, we visited David Davies' lab, my host at the NIH, to examine a large 3D model of chymotrypsin. Sili circled the model, probing every angle silently, seeing connections and patterns others missed. That moment sparked a collaboration spanning four decades, one that helped transform our understanding of AChE.

Attempts to determine the 3D structure of AChE date back to 1967, when Leuzinger and Baker first crystallized the 11S tetrameric form of the enzyme from the electric eel, *Electrophorus electricus* (Leuzinger & Baker, 1967). However, these early crystals were problematic, as later studies revealed. Working with his student Lili Anglister, Sili showed that the 11S tetramer possessed proteolytic “nick” sites, a likely reason why the best crystals diffracted only asymmetrically to modest resolutions (Anglister & Silman, 1978). Additionally, their work revealed that the native elongated forms of AChE possess a distinctive structure: a multi‐globular head composed of tetramers of the catalytic subunits, linked to a collagen‐like tail. This tail anchors AChE to the basal lamina within the synaptic cleft at the neuromuscular junction, facilitating its optimal physiological function.

Recognizing the need for a better crystallization candidate, Sili focused on the dimeric form of AChE from the electric organ of the electric ray, *Torpedo californica*. With his student Tony Futerman, he developed a mild purification method involving phosphatidylinositol‐specific phospholipase C (PIPLC) treatment, yielding a highly purified, water‐soluble preparation suitable for crystallization (Futerman et al., 1985; Sussman et al., 1988). This breakthrough paved the way for successful structural studies of the enzyme.

In collaboration with close colleagues at the Weizmann Institute, including Lilly Toker, Michal Harel, and Felix Frolow, as well as visiting scientists Adrian Goldman and Christian Oefner, we determined the structure of AChE (PDB_ID 2ace) in a landmark 1991 *Science* publication (Sussman et al., 1991). The results were astonishing; rather than being located in a negatively charged cleft near the surface, as had been widely expected, the active site was found to be deeply buried at the base of a narrow gorge lined with conserved aromatic residues.

A visually striking video, featuring Sir John Kendrew,* produced at the Weizmann Institute, vividly illustrates this groundbreaking discovery. The elucidation of the AChE structure has had a profound impact, revolutionizing the fields of synaptic transmission, drug design, Alzheimer's disease research, and the development of antidotes against toxins and nerve agents.

The unique structural features of AChE raised compelling questions about how substrates and products traverse the deeply buried active site. In response, Sili and colleagues proposed the “back‐door” hypothesis: that reaction products and/or solvent molecules might exit through transient openings adjacent to the active site at the base of the gorge (Figure 1) (Axelsen et al., 1994; Gilson et al., 1994; Ripoll et al., 1993). Nearly two decades later, in collaboration with teams in Grenoble and Shanghai, a combination of x‐ray crystallography and molecular dynamics simulations was used to identify in detail the structural elements comprising the proposed “back door” (Sanson et al., 2011; Xu et al., 2010) This elusive exit was found to lie between two subdomains of the *Tc*AChE monomer, which had been characterized in earlier work (Morel et al., 1999).

**FIGURE 1:**
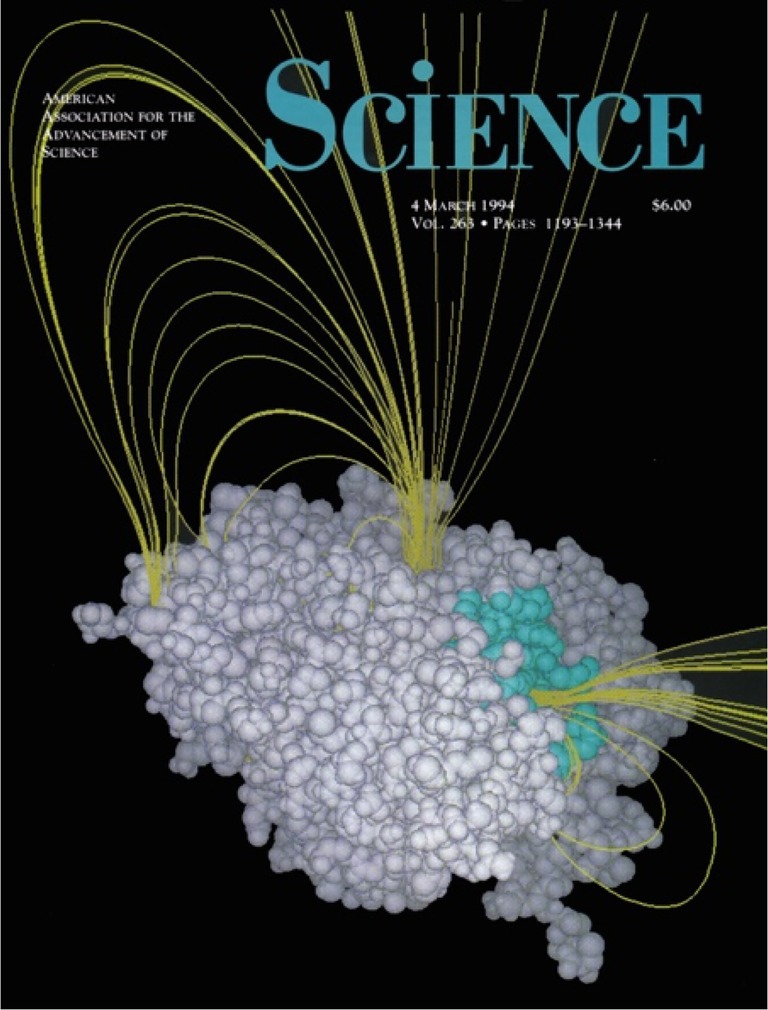
The cover of *Science*, March 4, 1994 (Gilson et al., 1994), showing the “back door” in *Tc*AChE. Eleсtrostatic lines of force (yellow) emanate from the interior of the active site (buried). The field lines exit mostly through the main entrance (blue atoms) or through a channel (“back door”) to the active site.

Sili's fascination with AChE was unrelenting. He investigated its anchoring, folding, localization, and evolution, building an extraordinarily detailed molecular picture through collaborations worldwide (Figures 2 and 3). He was not only a protein chemist and neurobiologist but also became a structural biologist, always attentive to fit, density, and the value of the R‐free.

**FIGURE 2:**
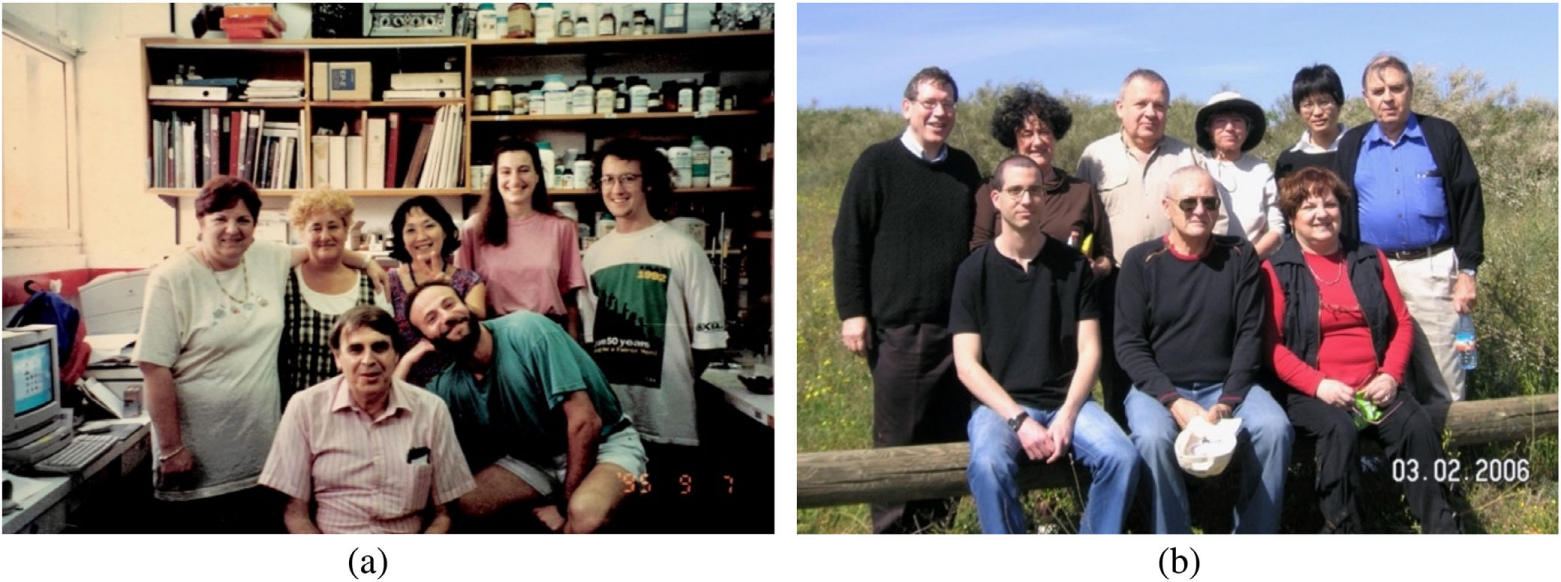
Israel Silman with some of his students and colleagues (a) September 1995 at the Weizmann Institute. (Top row, left to right) Esther Roth, Lilly Toker, Irina Shin, Mia Raves, Kurt Giles; (bottom row, left to right) Israel Silman and David Kreimer (b) March 2006 on a trip to the Soreq Forest (Top row, left to right) Joel Sussman, Lilly Toker, Lev Weiner, Michal Harel, Yechun Xu, and Israel Silman (bottom row, left to right) Aviv Paz, Yacov Ashani, and Esther Roth.

**FIGURE 3:**
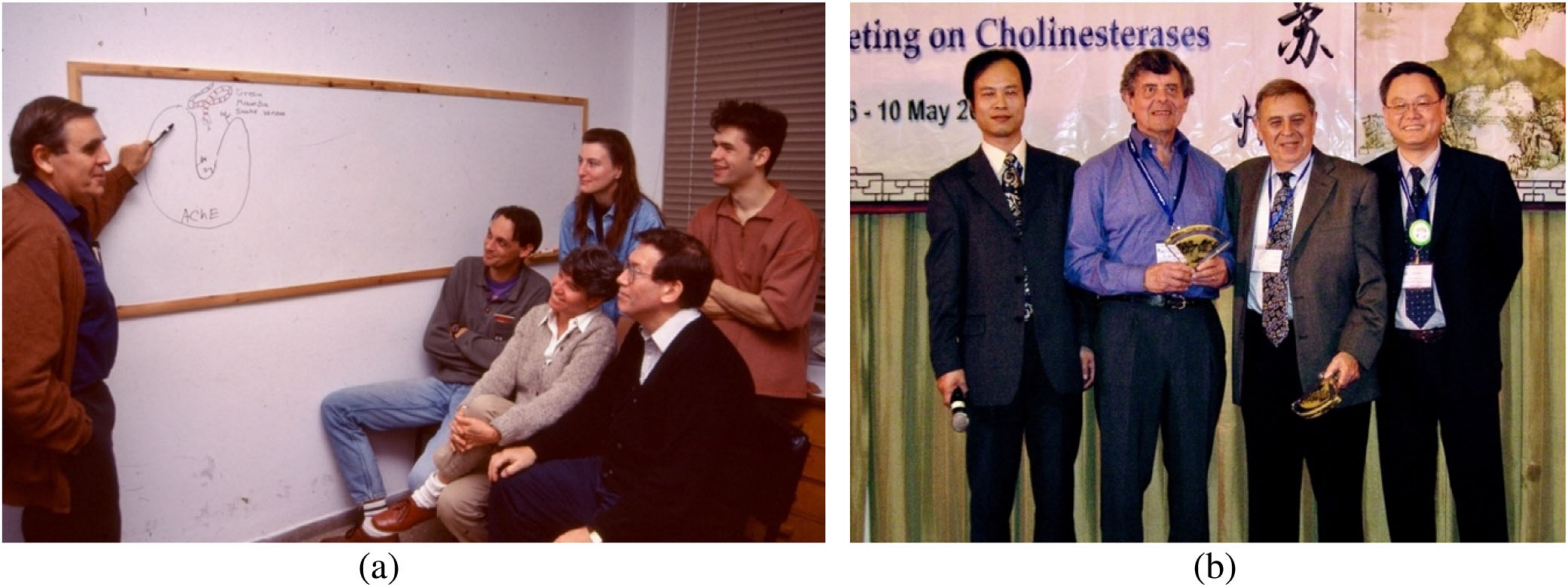
Israel Silman with colleagues in Israel and abroad (a) Sili discussed with students and colleagues how the Green Mamba snake toxin, fasciculin‐II (FAS‐II), binds and blocks AChE's activity (Harel et al., 1995), in December 1995 at the Weizmann Institute. (On the Left), Israel Silman; (top row, left to right) Mia Raves and Raimond Ravelli, (bottom row, left to right) Gerard Kleywegt, Michal Harel, and Joel Sussman; (b) Sili with his close colleagues at the 9th International Meeting on Cholinesterases in Suzhou, China in May 2007 (from Left to right) Hualiang Jiang, Jean Massoulié, Israel Silman, and Karl Tsim.

One of his most intriguing projects was studying X‐ray‐induced damage in proteins. Preparatory work for time‐resolved measurements on AChE revealed that synchrotron radiation caused highly specific chemical damage, including selective disulfide bond breakage. Studies with Martin Weik, Raimond Ravelli, and Gitay Kryger had profound implications for structural biologists working with bright synchrotron sources. The visualization of the breaking of a particular disulfide bond can be seen dramatically in an animation,† movie,‡ and the cover of the *PNAS* (Weik et al., 2000) issue in which the paper appeared (Figure 4).

**FIGURE 4:**
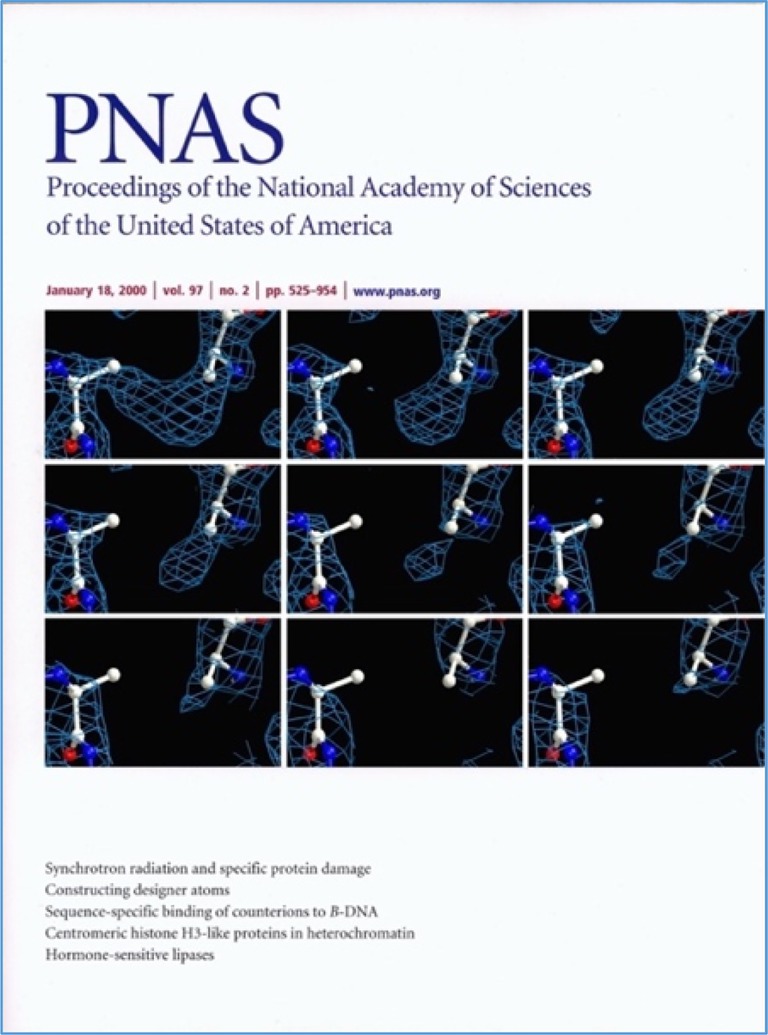
The cover of *PNAS* January 16, 2000, shows the specific damage synchrotron radiation can cause to a protein structure, even at cryogenic temperatures (Weik et al., 2000), (PDB_IDs: 1qid, 1qie, 1qif, 1qig, 1qih, 1qii, 1qij, 1qik, and 1qim).

Sili's talents extended further. He explored the role of intrinsically disordered proteins (IDPs) with three gifted students, Tzviya Zeev‐Ben‐Mordehai, Aviv Paz, and Simone Botti, showing that the cytoplasmic domains of cholinesterase‐like adhesion molecules were intrinsically disordered (Paz et al., 2008; Zeev‐Ben‐Mordehai et al., 2003), a property that he realized is key to their function. This insight led to his co‐organizing the first international conference on IDPs in Budapest in 2007 (Figure 5).

**FIGURE 5:**
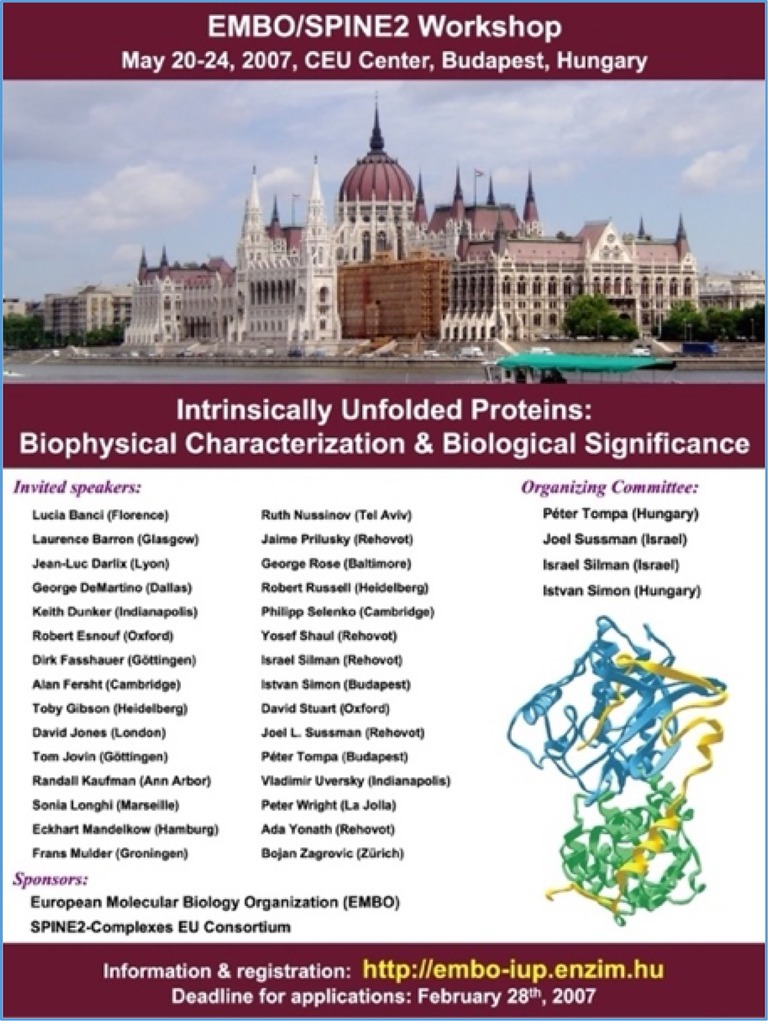
Poster for the International Conference on IDPs, Budapest, May 2007.

Sili published 17 papers in *Protein Science*, two of which were featured on the cover (Figure 6). His most recent publication, also the last paper published in his lifetime (Lushchekina et al., 2024), was a fascinating study investigating an unusual site in AChE containing four conserved aspartic acid residues. The study combined meticulous biochemical and crystallographic analyses with critical *in silico* work carried out by Sofya Lushchekina, who recently came to the Weizmann Institute from Russia due to the war in Ukraine.

**FIGURE 6:**
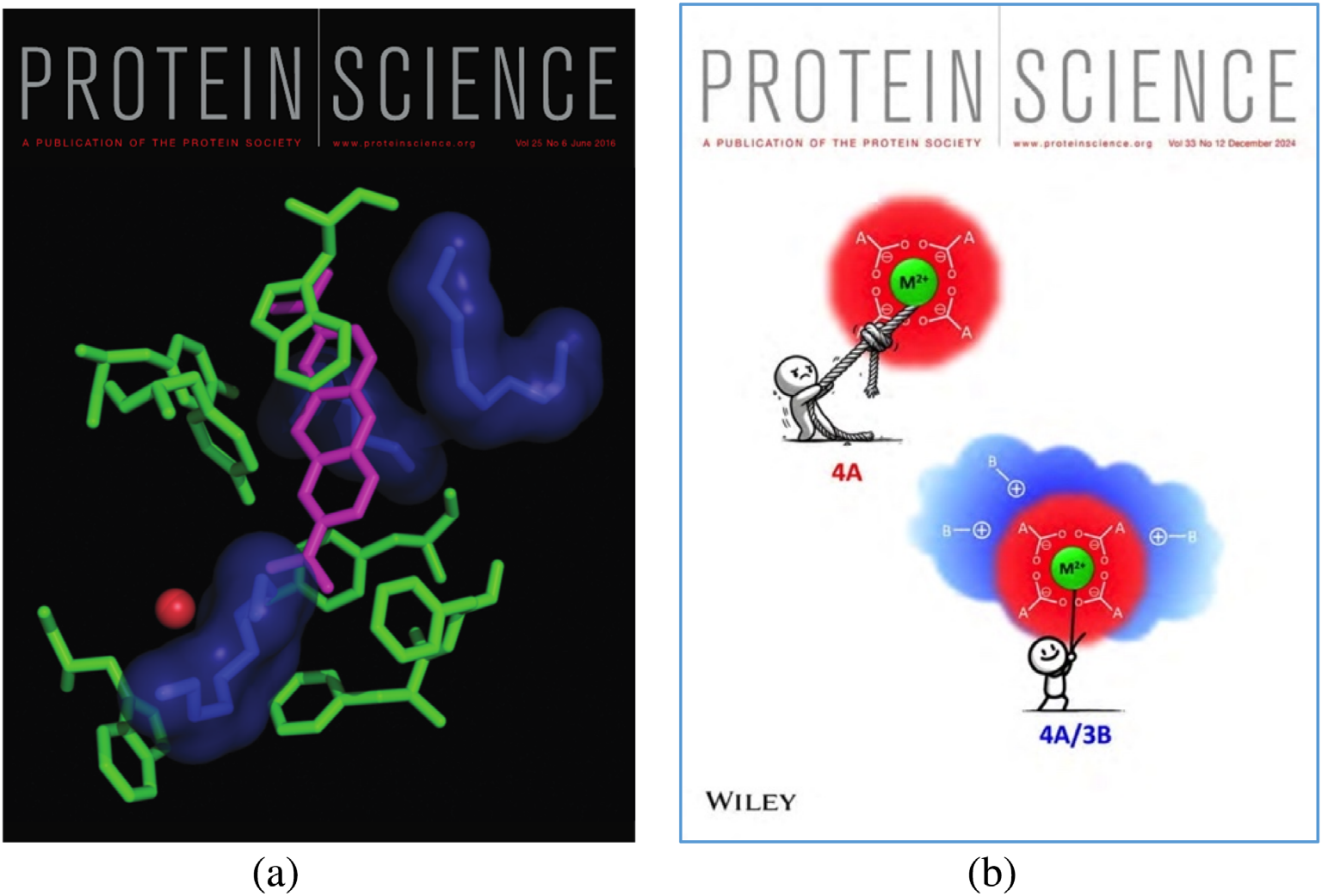
Covers of *Proteins Science* issues featuring two of Silis' papers. (a) Impact of PEGs on the positioning of the ligand in the crystal structure of the methylene blue/acetylcholinesterase complex (Dym et al., 2016), (PDB_IDs: 5e4t, 5dlp, 5e2i, 5e4j); (b) For a divalent cation, it is quite hard (takes significant energy) to unbind it from structural motifs composed of four acidic residues (4A). However, it is much easier in motifs where three surrounding basic residues form ion pairs with three carboxylates (4A/3B). In contrast to the 4A motif, the 4A/3B motif doesn't undergo a conformational change upon binding or unbinding the divalent cation (Lushchekina et al., 2024).

Even during the COVID‐19 pandemic, Sili continued mentoring young scientists. Together, we co‐led a remote mentoring project for undergraduates from Tsinghua University, Guangdong Technion Israel Institute of Technology, and Shanghai Jiao Tong University, China. This student project evolved into a 2‐year international collaboration on orphan proteins. It culminated in a cover‐featured paper in *Proteins* (Liu et al., 2023) (Figure 7), a testament to Sili's enduring dedication to nurturing the next generations.

**FIGURE 7:**
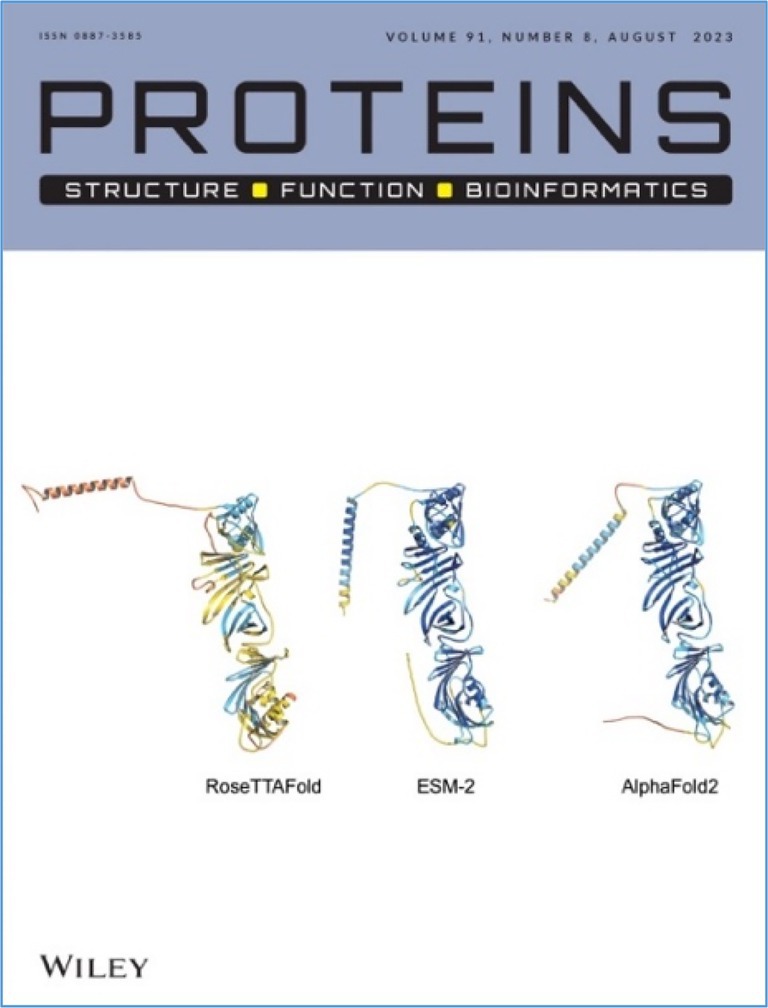
Cover of *Proteins*, August 2023, showing the predicted models of an orphan nematode protein using three different AI/Deep Learning algorithms (Liu et al., 2023).

Upon hearing of his passing, two of the co‐author students from China shared heartfelt tributes:

“Prof. Silman was not only a brilliant scientist but also an exceptional mentor and advisor. His influence on my journey will always stay with me.”—Jing Liu, now a PhD student at the Weizmann Institute.

“His wisdom, kindness, and contributions will be remembered. I feel truly fortunate and honored to have learned from him.”—Rongqing Yuan, now a PhD student at the UT Southwestern Medical Center.

Sili leaves a legacy that transcends his groundbreaking discoveries. He trained and inspired generations of scientists across Israel, Europe, Asia, and the Americas. Many of his former students have advanced in their careers, for example, Jerry Eichler—Chair, Dept. of Life Sciences, Ben Gurion Univ.; Yadin Dudai—Dean, Faculty of Biology, Weizmann Institute; Gabi Amitai—Distinguished Scientist at the Israel Institute of Biological Research.

At the Weizmann Institute, he fostered not only scientific excellence but a culture of humility, intellectual joy, and generosity. Deeply committed to the world beyond science, Sili used his voice to defend human dignity and inspire hope.

Sili was a true Renaissance man, with a deep love of classical and jazz music, fine cuisine, and excellent wines (Figure 8). His broad knowledge and passion for art, literature, and theater made him a fascinating conversationalist. He was married to Aliza, with whom he had a son, Jonathan Silman.

**FIGURE 8:**
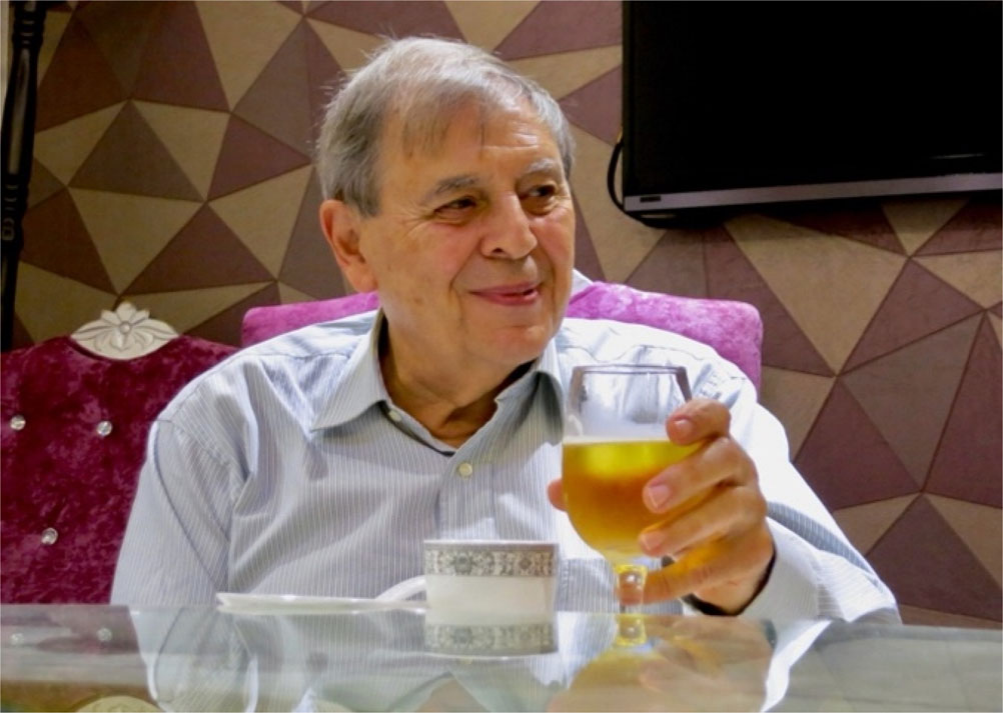
Israel Silman in Shanghai (May, 2013) sampling some fine Chinese wine after the fascinating Meeting on Cholinergic Mechanisms in Hangzhou.

For me, he was far more than a collaborator; he was a mentor, confidant, and dear friend. His mind was restless, imaginative, and precise. He will be deeply missed and long remembered.

## AUTHOR CONTRIBUTIONS

**Joel L. Sussman:** Conceptualization; writing – original draft.

## Footnotes

*
https://www.youtube.com/watch?v=ecdGVoboe44.

†
https://www.weizmann.ac.il/CSB/Sussman/radiation-damage-0 Animation by Gitay Kryger showing the breaking of a specific disulfide bond by X‐ray radiation.

‡
https://www.youtube.com/embed/8jDguCM10no Movie on The Surprising Nature of Synchrotron Radiation by Richard Gillilan.
